# Involvement of Nuclear Receptors PPAR-α, PPAR-γ, and the Transcription Factor Nrf2 in Cellular Protection Against Oxidative Stress Regulated by H_2_S and Induced by Hypoxia–Reoxygenation and High Glucose in Primary Cardiomyocyte Cultures

**DOI:** 10.3390/antiox14040482

**Published:** 2025-04-17

**Authors:** Luz Ibarra-Lara, Araceli Sánchez-López, Leonardo del Valle-Mondragon, Elizabeth Soria-Castro, Gabriela Zarco-Olvera, Mariana Patlán, Verónica Guarner-Lans, Juan Carlos Torres-Narváez, Angélica Ruiz-Ramírez, Fernando Díaz de León-Sánchez, Víctor Hugo Oidor-Chan, Vicente Castrejón-Téllez

**Affiliations:** 1 Department of Pharmacology, National Institute of Cardiology Ignacio Chávez, Mexico City 14080, Mexico; luz.ibarra@cardiologia.org.mx (L.I.-L.); leonardo.delvalle@cardiologia.org.mx (L.d.V.-M.); gabriela.zarco@cardiologia.org.mx (G.Z.-O.); juan.torres@cardiologia.org.mx (J.C.T.-N.); 2 Department of Pharmacobiology, Center for Research and Advanced Studies of the National Polytechnic Institute, Mexico City 07360, Mexico; asanchezl@cinvestav.mx; 3 Department of Cardiovascular Biomedicine, National Institute of Cardiology Ignacio Chavez, Mexico City 14080, Mexico; elizabeth.soria@cardiologia.org.mx (E.S.-C.); angelica.ruiz@cardiologia.org.mx (A.R.-R.); 4 Subdirectorate of Basic and Technological Research, National Institute of Cardiology Ignacio Chávez, Mexico City 14080, Mexico; mariana.patlan@cardiologia.org.mx; 5 Department of Physiology, National Institute of Cardiology Ignacio Chavez, Juan Badiano 1, Sección XVI, Tlalpan, Mexico City 14080, Mexico; veronica.guarner@cardiologia.org.mx; 6 Laboratory of Post-harvest of Plant Genetic Resources and Natural Products, Department of Health Sciences, Autonomous Metropolitan University, Iztapalapa Campus, Mexico City 09310, Mexico; fdls@xanum.uam.mx; 7 Department of Biotechnology, Autonomous Metropolitan University, Iztapalapa Campus, Av. Ferrocarril San Rafael Atlixco 186, Leyes de Reforma, Iztapalapa, Mexico City 09310, Mexico

**Keywords:** sodium hydrosulfide (NaHS), hypoxia–reoxygenation, hyperglycemia, PPAR-α, PPAR-γ, antioxidant capacity

## Abstract

Myocardial oxidative stress increases under conditions of hyperglycemia and ischemia/reperfusion (I/R) injury, leading to cellular damage. Inhibition of oxidative stress is involved in the cardioprotective effects of hydrogen sulfide (H_2_S) during I/R and diabetes, and H_2_S has the potential to protect the heart. However, the mechanism by which H_2_S regulates the level of cardiac reactive oxygen species (ROS) during I/R and hyperglycemic conditions remains unclear. Therefore, the objective of this study was to evaluate the cytoprotective effect of H_2_S in primary cardiomyocyte cultures subjected to hyperglycemia, hypoxia–reoxygenation (HR), or both conditions, by assessing the PPAR-α/Keap1/Nrf2/p47phox/NOX4/p-eNOS/CAT/SOD and the PPAR-γ/PGC-1α/AMPK/GLUT4 signaling pathways. Treatment with NaHS (100 μM) as an H_2_S donor in cardiomyocytes subjected to hyperglycemia, HR, or a combination of both increased cell viability, total antioxidant capacity, and tetrahydrobiopterin (BH_4_) concentrations, while reducing ROS production, malondialdehyde concentrations, 8-hydroxy-2′-deoxyguanosine, and dihydrobiopterin (BH_2_) concentrations. Additionally, the H_2_S donor treatment increased the expression and activity of PPAR-α, reversed the reduction in the expression of PPAR-γ, PGC-1α, AMPK, GLUT4, Nrf2, p-eNOS, SOD, and CAT, and decreased the expression of Keap1, p47phox and NOX4. Therefore, the treatment with the H_2_S donor protects cardiomyocytes from damage caused by hyperglycemia, HR, or both conditions by reducing oxidative stress markers and improving antioxidant mechanisms, thereby increasing cell viability and “cardiomyocyte ultrastructure”.

## 1. Introduction

Diabetes is a chronic disease characterized by hyperglycemia, which causes damage to various organs including the heart, vasculature, eyes, kidneys, and nerves. More than 90% of cases of diabetes in humans correspond to the type 2 diabetes (T2D) variety, a condition characterized by deficient insulin secretion by pancreatic β-cells, tissue insulin resistance (IR), and an inadequate compensatory insulin secretory response [1]. T2D leads to a two- to fourfold increase in mortality rates from heart disease and is associated with both microvascular and macrovascular complications. The latter include accelerated atherosclerosis, which leads to severe peripheral vascular disease and premature coronary artery disease (myocardial infarction, angina, etc.) [2,3].

Under hyperglycemic conditions, the imbalance between the cellular antioxidant system and the production of reactive oxygen species (ROS) generates oxidative stress, which leads to the development of T2D. The high production of ROS causes structural and functional modifications in proteins, lipids, and nucleic acids [4]. Processes such as myocardial ischemia/reperfusion (I/R) lead to high morbidity and mortality in humans and are also associated with T2D. Oxidative stress is one of the most important pathological mechanisms in I/R injury, causing apoptosis, autophagy, inflammation, and other cellular damage through multiple pathways. This leads to irreversible myocardial damage and ultimately cardiac dysfunction [5].

Increased oxidative stress during cardiac injury may be due to uncoupling of the mitochondrial respiratory chain, caused by inactivation of complex I [6]. However, the increase in ROS during cardiac injury may also be due to altered antioxidant capacity. This results from reduced activity of superoxide dismutase (SOD) and catalase (CAT) [7], uncoupling of endothelial nitric oxide synthase (eNOS) due to decreased tetrahydrobiopterin concentration [8], or stimulation of enzymes related to oxidation, including xanthine oxidase, cyclooxygenase, inducible nitric oxide synthase (iNOS), and NADPH oxidase (Nox). In fact, Nox4 in cardiomyocytes is a critical mediator of oxidative stress and cardiac dysfunction, being a major source of O_2_^−^ and H_2_O_2_ production in the heart [9]. The cellular antioxidant defense mechanism that regulates ROS production under normal physiological conditions involves enzymes such as glutathione peroxidase (GPx), SOD, CAT, glutathione reductase, and molecules such as vitamins A, E, and C, as well as minerals like Cu^2+^, Se^2+^, and Mn^2+^ [4].

Moreover, activation of peroxisome proliferator-activated receptor alpha (PPAR-α) reduces oxidative stress and improves ventricular ultrastructure and hemodynamics in myocardial ischemia without flow [10]. PPAR-α, binds to the promoter region of specific target genes described as PPAR response elements (PPREs) after heterodimerization with the retinoid X receptor (RXR), acting as a transcription factor. The coactivator-1 alpha of PPAR-γ (PGC1-α) plays an important role in gene transcription through its interaction with PPAR-α and the activation of the complex of these elements is a key factor in mitochondrial biogenesis activating several transcription factors, including the nuclear factor erythroid 2-related factor 2 (Nrf2) [11]. Under normal conditions, Nrf2 is constantly ubiquitinated by the Kelch-like ECH-associated protein 1 (Keap1) and degraded in the proteasome. Keap1 is inactivated after ROS exposure, and Nrf2 is phosphorylated. Phosphorylated Nrf2 (p-Nrf2) accumulates in the nucleus and binds to antioxidant response element (ARE) sites, subsequently activating many genes, including antioxidants, detoxifying enzymes, and transport molecules [12].

Sodium hydrosulfide (NaHS) is a hydrogen sulfide (H_2_S) donor, and H_2_S has traditionally been considered a toxic gas. However, the function of H_2_S as an endogenously generated biological mediator has recently been recognized. Perfusion with NaHS in an ex vivo isolated rat heart model protects the heart against I/R-induced arrhythmias. Additionally, the same study reported that incubation of rat cardiomyocytes with NaHS exposed to an ischemic solution improved cell viability and morphology [13]. Furthermore, NaHS treatment during reperfusion resulted in a significant improvement in cardiac function compared to the I/R group in isolated perfused rat hearts. This effect was attributed to the opening of the ATP-sensitive potassium (K_ATP_) channels expressed in cardiomyocytes [14]. In another investigation using aged cardiomyocytes, exogenous NaHS promoted the recovery of PC-induced cardioprotection by inhibiting the opening of the mitochondrial permeability transition pore (mPTP) through the activation of the ERK1/2-GSK-3β, PI3K-Akt-GSK-3β, and PKC-ε-mKATP pathways. In addition, post-conditioning (PC) lost its cardioprotective effects against hypoxia-reperfusion (HR) damage when animals were exposed to NaHS [15].

To date, no study has linked the activation of the PPAR-α/PPARγ/Keap1/Nrf2/p47phox/NOX4/p-eNOS/CAT/SOD signaling pathway in primary cardiomyocyte cultures incubated with high glucose concentrations and exposed to HR and then incubated with the H_2_S donor (NaHS). Therefore, the aim of this research was to study the protective effect of NaHS in cardiomyocytes incubated under hyperglycemic conditions, HR, or both, evaluating the aforementioned signaling pathway, cell viability, total antioxidant capacity, ROS production, quantification of malondialdehyde, 8-hydroxy-2′-deoxyguanosine, and tetrahydrobiopterins.

## 2. Materials and Methods

### 2.1. Animals

Wistar rats, both female and male, aged from 1 to 3 days post birth, were used. The animals were provided by the animal facility of the National Institute of Cardiology Ignacio Chavez, Mexico, and the protocol was carried out following the guidelines of the institutional ethics committee, protocol number INC/CICUAL/010/2024, and the Official Mexican Standard with experimental animals were carried out following Federal Stanards, Secretaría de Agricultura, SAGARPA, NOM-062-ZOO-1999, México.

### 2.2. Neonatal Rat Cardiomyocytes (NRCMs) Isolation and Culture

NRCMs were isolated from 2–3-day-old Wistar rats as previously described [16]. The excised hearts were minced, and the ventricles were digested four times in trypsin (0.25% Invitrogen, Carlsbad, CA USA) in a sterile environment for 15 min. NRCMs were cultured in the medium (F-10 (1X) nutrient mixture (HAM) (+) containing L-glutamine (Gibco, Waltham, MA, USA), 5.5 mmol/L D-glucose, and supplemented with 10% heat-inactivated fetal bovine serum (FBS, Invitrogen, Carlsbad, CA, USA), 100 U/mL of penicillin and 100 mg/L of streptomycin (Gibco, Waltham, MA, USA). NRCMs (1 × 10^6^) were placed in six-well culture plate and incubated at 37 °C in a humidified atmosphere (5% CO_2_/95% O_2_). Experiments were performed on beating and confluent monolayers from the 3rd to 5th day of culture. Initially, cardiomyocytes were exposed to vehicle (phosphate saline buffer (PBS)) prior to treatment with NaHS, 100 μM.

Anaerobic bags (GasPack™ EZ system, BD Biosciences. Becton Dickinson Pty Ltd. 4 Research Park Drive, Macquarie University Research Park North Ryde, NSW 2113, Australia) were used to induce hypoxia in cultured cardiomyocytes [17,18]. Six-well plates containing cultured cardiomyocytes were exposed for 2 h to an atmosphere composed of 95% N_2_ and 5% CO_2_ in a sealed bag containing an oxygen-consuming palladium catalyst, creating a hypoxic environment (25–35 mmHg PO_2_). Immediately after the hypoxia period, cell cultured plates were placed in a standard incubator for reoxygenation for 1 h before further assays [19,20]. The success of the induction of hypoxia was evaluated through the increased expression of HIF-1α by Western blot.

Cell were cultured in F-10 medium containing 25 mmol/L of glucose for 48 h [21] for the high-glucose treatment. To explore the role of glucose hyperosmolarity, cells were exposed to mannitol (19.5 mM), (D-mannitol, Sigma-Aldrich, St. Louis, MO, USA). The treatment with vehicle (PBS) and NaHS (100 μM) were administered 1 h before the culture was subjected to HR. This treatment was administered after subjecting the cells to HG.

**Figure 1:**
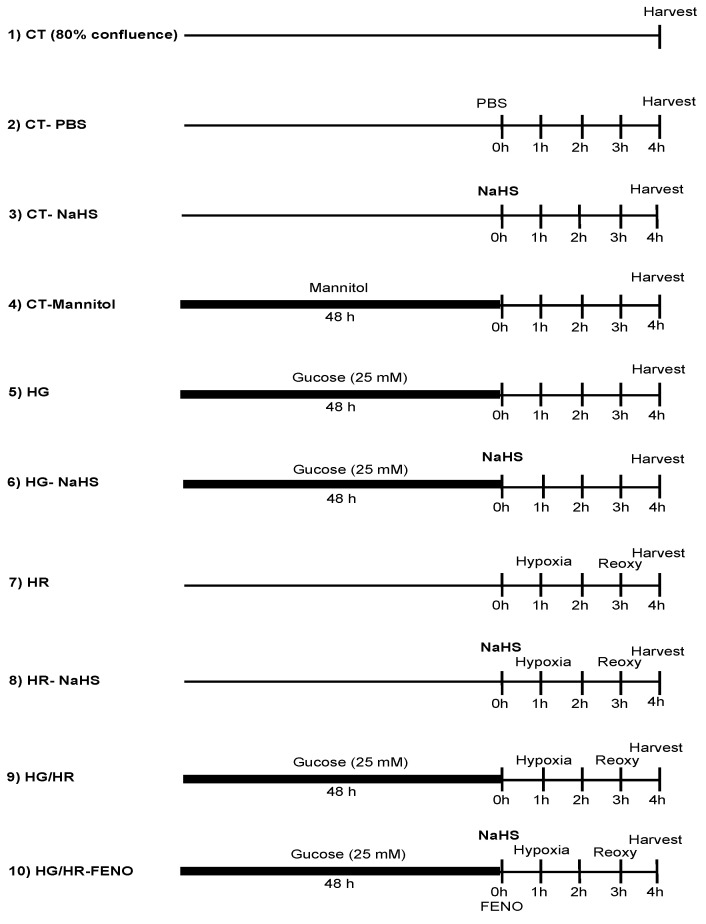
Experimental groups in the current study. Mannitol (19.5 mM) and high glucose (25 mM) were administered 48 h before NaHS (100 μM). The treatment with NaHS lasted for 4 h; CT = control, HR = hypoxia/reoxygenation, HG = high glucose.

Cell cultures were divided into the following experimental groups: control (CT), high glucose (HG); and hypoxia/reoxygenation (HR); group 1: CT, group 2: CT/PBS, group 3: CT/NaHS (100 µM), group 4: CT/Mannitol, group 5: HG (25 mM), group 6: HG/NaHS (100 μM), group 7: HR, group 8: HR/NaHS (100 μM) group group 9: HG/HR, group 10: HG/HR NaHS (100 μM) (Figure 1).

### 2.3. HIF1-α Expression

We assessed the expression of the hypoxia-inducible factor (HIF-α) to determine if the method used to produce hypoxia via the GasPack™ EZ system was effective. This determination was made by Western blot. HIF-α activates genes that encode for proteins responsible for increasing oxygen availability and enabling metabolic adaptation in the absence of oxygen, thus controlling the expression of numerous gene products and proteins [22].

### 2.4. Cell Viability

Cell viability was determined according to Strober W and Crowley LC [23,24]. A volume of 100 μL 0.4% Trypan blue was added to 1 mL of cells (1 × 10^6^). An aliquot of 50 μL of the cell suspension was loaded to a Neubauer chamber (Neubauer, Marienfeld, 0.0025 mm^2^ Wollerspfad Lauda-Konigshofen, Germany) and immediately examined under a microscope at 10× magnification. The numbers of alive (unstained) and dead (blue) cells were counted. A cell viability of 95% was established to consider the culture in the healthy logarithmic phase.

### 2.5. Antioxidant Capacity Assay

Total antioxidant capacity was determinate using the method described by Apak. et al. [25]. A suspension of 6 × 10^6^ cells from different experimental groups was evaluated. The cells were centrifuged 1500 rpm for 10 min, then diluted with 145 μL of 0.1 M phosphate buffer at pH 7.5 and shaken at 500 rpm for 200 s, and 100 μL of the diluted sample was treated with 50 μL of 0.01 M CuCl_2_ and shaken at 500 rpm for 200 s. Then, of 0.01 M bathocuproine was added and the sample was vortexed again at 500 rpm for 200 s. The concentration of Cu^2+^ reduced to Cu^+^ was measured using a spectrometer at 490 nm (DW2000, SLM-Aminco, Urbana, IL, USA). Total antioxidant capacity is expressed as μmol/L of Cu^2+^ reduced to Cu^+^.

### 2.6. ROS Production

The medium was removed from cells from the different treatments (1 × 10^6^), and the cells were washed with PBS and incubated for 30 min with CellRox™ Green Reagent (ThermoFisher Scientific, Waltham, MA, USA) at a final concentration of 5 µM/mL. The was process performed in the dark as much as possible. After incubation, the medium was removed, and cells were washed twice with PBS. Finally, the cells were scraped off with 1 mL of PBS and placed in dark Eppendorf tubes to avoid exposure to light. The fluorescence emitted by the interaction of free radicals and the CellRox indicator was determined by flow cytometry, using a BD FACSAria Fusion Flow Cytometer (Becton Dickinson, Mountain View, CA, USA) and the FlowJo10.8.1 software. The results were calculated as the geometric mean fluorescence (MF) of 5000 events, obtained by region and its fluorescence signal, observed as a displacement of the fluorescence depending on the treatment in each cell group loaded with the CellRox indicator (Life Technologies Corporation, Willow Creek Road, Eugene, OR, USA). Results were compared with the intrinsic fluorescence of a group of cells that were not incubated with the indicator [26].

### 2.7. Quantification of Malondialdehyde (MDA)

Malondialdehyde was determined by capillary zone electrophoresis in a cardiomyocyte suspension (6 × 10^6^ cells) from the different experimental groups, as described Sánchez A [27]. The sample was deproteinized with cold methanol in a 1:1 ratio, centrifuged at 16,000× *g* for 15 min, and filtered with 0.22 µm nitrocellulose membrane filters (Millipore, Billerica, MA, USA); it was then diluted 1:10 with 0.1 M cold sodium hydroxide and analyzed. The P/ACE™ MDQ Capillary Electrophoresis System (Beckman Coulter, Brea, CA, USA) was used for this purpose. The samples were injected under hydrodynamic pressure at 0.5 psi for 10 s. Separation was performed at −25 kV for 4 min at 267 nm. The capillary was washed between runs with 0.1 M NaOH for 2 min, distilled water for 2 min, and buffer for 4 min. The concentration of MDA is expressed in μM and was determined using a standard curve.

### 2.8. Quantification of 8-Hydroxy-2′-Deoxyguanosine (8-OH-2dG)

8-OH-2-dG was determined by capillary zone electrophoresis and UV detection with diode array detection, as described Sánchez A. et al. [27]. The myocyte suspension sample (6 × 10^6^ cells) from different experimental groups was deproteinized with 20% trichloroacetic acid, in a 10:1 ratio. It was centrifuged at 16,000× *g* for 15 min and filtered with 0.22 µm nitrocellulose membrane filters. The samples were analyzed using the P/ACE™ MDQ Capillary Electrophoresis System (Beckman Coulter, CA, USA). The capillary was preconditioned by passing 2 M solution of sodium hydroxide for 30 min, followed by deionized water for 30 min, and then the run buffer (10 mM borates at pH 9.0) for 30 min. The sample was injected under hydrodynamic pressure at 0.5 psi for 10 s. The separation was carried out at 20 kV for 8 min at 200 nm. The capillary was washed between runs with 2 M sodium hydroxide for 2 min and distilled water for 2 min. The results are expressed in pmoles/mL. The concentration of 8-OH-2dG was determined using a standard curve. Injection conditions were adapted from Kvasnicova et al. [28].

### 2.9. Capillary Zone Electrophoresis for Determination of BH_4_ and BH_2_

The myocyte suspension sample (6 × 10^6^ cells) from the different experimental groups was evaluated as described Ibarra-Lara. et al. [10]. Briefly, 50 μL of sample containing 6 × 10^6^ cells were deproteinized with cold methanol (1:1 *v*/*v*), centrifuged at 16,000× *g* for 15 min al 10 °C, and filtered with 0.22 μm nitrocellulose membrane (Millipore, Billerica, MA, USA). Measurement was performed using a P/ACE™ MDQ Capillary Electrophoresis System (Beckman Coulter, Mexico City, Mexico), with laser-induced fluorescence detection. Data are expressed as pmol/mg of protein for BH_4_ and BH_2_.

### 2.10. Palmitoyl CoA Oxidase Activity

This study [29] reports the development of a simple, specific, and highly sensitive fluorometric assay for peroxisomal fatty acyl-CoA oxidase activity. In this procedure, fatty acid acyl-CoA-dependent H_2_O_2_ production was coupled in a peroxidase-catalyzed reaction to the oxidation of scopoletin (6-methoxy-7-hydroxycoumarin), a highly fluorescent compound, to a non-fluorescent product.

Peroxisomal palmitoyl CoA oxidase activity was determined as previously described [29]. Cardiomyocyte cultures (6 × 10^6^) were diluted with 0.25 M sucrose, 1 mM EDTA, and 0.1%. ethanol. Samples containing 500 µg of protein were incubated at 37 °C for 30 min with constant shaking in the reaction mixture containing 60 mM Tris–HCl (pH 8.3), 35 µM palmitoyl CoA, 50 µM FAD, 1 µM scopoletin, peroxidase (3 units), 0.6 mg bovine serum albumin, and triton X-100 (0.01%) to a final volume of 1 mL The reaction was stopped by the addition of 4 mL of 0.1 M borate buffer (pH 10). Fluorescence was measured at 470 nm emission and 395 nm excitation with a Varian Cary Eclipse Fluorescence Spectrophotometer (Cary Eclipse, Varian Co., Mulgrave, Victoria, Australia) Data are expressed as nmol scopoletin per mg of protein for 30 min.

### 2.11. Protein Expression by Western Blot

The total protein content in the cell cultures was quantified as described in a previous study [27]. Protein extracts (80 µg) from cell lysates were separated using a 12% SDS–PAGE gel at 100 V for 2 h. Following electrophoresis, the proteins were transferred to a 0.45 µm polyvinylidene fluoride (PVDF) membrane (Millipore, Billerica, MA, USA) at 1 Å for 1 h. The membrane was then blocked with 5% Blotto, not-fat dry milk (Santa Cruz Biotechnology, Inc., Dallas, TX, USA) in PBS containing 0.1% Tween 20, as previously reported. The blots were incubated with primary antibodies: β-actin (1:5000, sc-47778), HIF1α (1:100, sc-53546), SOD Cu^2+/^Zn^2+^ (1:100, sc-17767), SOD Mn^2+^ (1:100, sc-137254), Catalase (1:100, sc-271803), p-NOS3 (Ser1177) (1:100, sc-12972), NOX4 (1:100, sc-518092), p47phox (1:100, sc-17845), Nrf2 (1:100, sc-365949), KEAP (1:100, sc-365626), PPAR-α (1:50, sc-398394), PPAR-γ (1:100, sc-7273), PGC-1α (1:100, sc-517380), AMPK (1:100, sc-398861), GLUT4 (1:100, sc-53566). All antibodies were obtained from Santa Cruz Biotechnology, Santa Cruz, CA, USA. After washing, the blots were probed with chemiluminescent HRP substrate (Immobilon Western, Millipore, Billerica, MA, USA), and the signals were detected by chemiluminescence. Densitometric analysis was performed using Quantity One 1D software Version 4.6.8, PC (Bio-Rad Laboratories Inc., Hercules, CA, USA). Blots were stripped and re-incubated with β-Actin antibody as a loading control. Bands densities are expressed as arbitrary units.

### 2.12. Mitochondria Ultrastructure

Mitochondrial ultrastructure in cardiomyocytes from the different experimental groups was examined using the method described by González-Morán [30]. Cardiomyocytes were fixed with 2.5% glutaraldehyde for 1 h, then stored overnight in a 0.1 M cacodylate buffer. After post-fixation with 0.1 M osmium tetroxide in cacodylate buffer, the samples were dehydrated in an ethanol gradient and embedded in EPON 812. Ultrathin sections (approximately 60 nm thick) were cut using a Leica Ultracut microtome and mounted onto copper grids. The sections were contrasted with uranyl acetate and examined under a JEM-1011 transmission electron microscope (JEOL Ltd., Tokyo, Japan) at 60 kV. Images of cardiomyocytes from each experimental group were taken randomly and evaluated at a magnification of 12,000×.

### 2.13. Statistical Analysis

Results are expressed as the mean ± standard error of the mean (SEM) from 3–6 independent experiments. Comparisons between two groups were performed using unpaired Student’s *t*-test. For multiple comparations, a two-way analysis of variance (ANOVA) followed by a Tukey post-hoc test was used (Sigma Plot 13 software, version 13.0 for windows). Statistical significance was set at *p* < 0.05.

## 3. Results

### 3.1. Evaluation of the Hypoxia–Reoxygenation (HR) Model in Primary Cardiomyocyte Cultures

**Figure 2:**
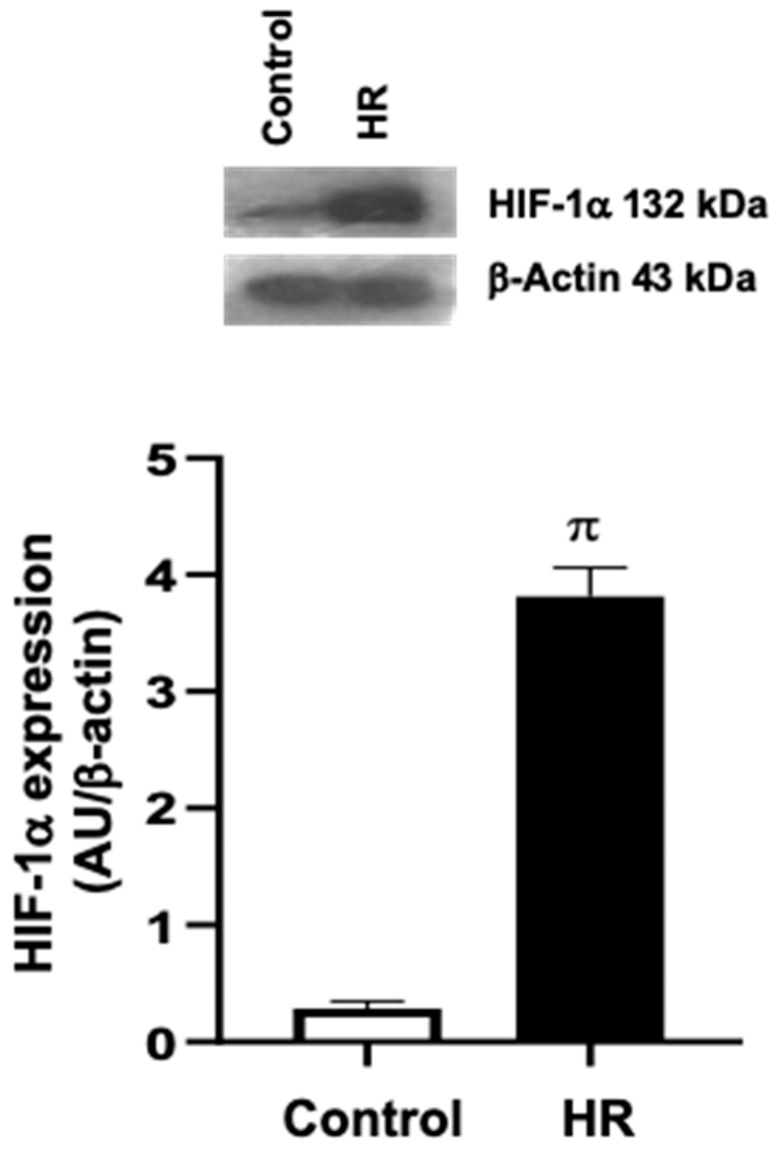
Expression of hypoxia-inducible factor 1α (HIF-1α) in primary cardiomyocyte cultures exposed to hypoxia–reoxygenation (HR). HR led to an increased in the expression of HIF-1α in cardiomyocytes. π = *p* < 0.05 vs. Control; *t*-test; *n *= 3.

Hypoxia in primary cardiomyocyte cultures was induced using anaerobic bags, followed by reoxygenation of the cells. To assess the effectiveness of our experimental protocol, the expression of HIF-1α was evaluated by Western blot. Cardiomyocytes exposed to HR conditions showed an increased expression of HIF-1α compared to the control group (Figure 2).

### 3.2. Evaluation of Cell Viability

**Figure 3:**
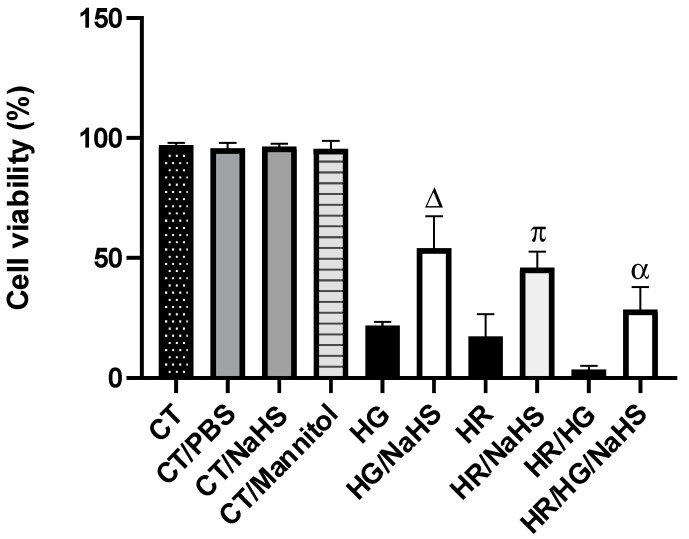
Evaluation of cell viability using the Trypan blue in primary cardiomyocyte cultures. Results from cells incubated with NaHS (100 μM), subjected to high glucose (HG), hypoxia–reoxygenation (HR), or both conditions (HG/HR) are shown. The graph represents the cell viability of the different experimental groups: CT, CT/PBS, CT/NaHS, CT/mannitol, HG, HG/NaHS, HR, HR/NaHS), HG/HR, and HR/HG7NaHS. Δ = *p* < 0.05 vs. HG; π = *p* < 0.05 vs. HR; α = *p* < 0.05 vs. HG/HR. Two-way ANOVA followed by a Tukey post hoc test; *n* = 6.

Cell viability in primary cardiomyocytes cultures from different experimental groups was evaluated using Trypan blue. Control cardiomyocytes incubated with PBS, NaHS, and mannitol showed an average cell viability of 95%. Cardiomyocytes exposed to hyperglycemic (HG), hypoxia–reoxygenation (HR), or HG/HR conditions exhibit a significant reduction in cell viability to 14%. However, incubation of the experimental groups (HG, HR, and HG/HR) with NaHS (100 μM) reversed the observed cell damage, resulting in a 42% increase in viability (Figure 3).

### 3.3. Total Antioxidant Capacity (TAC)

**Figure 4:**
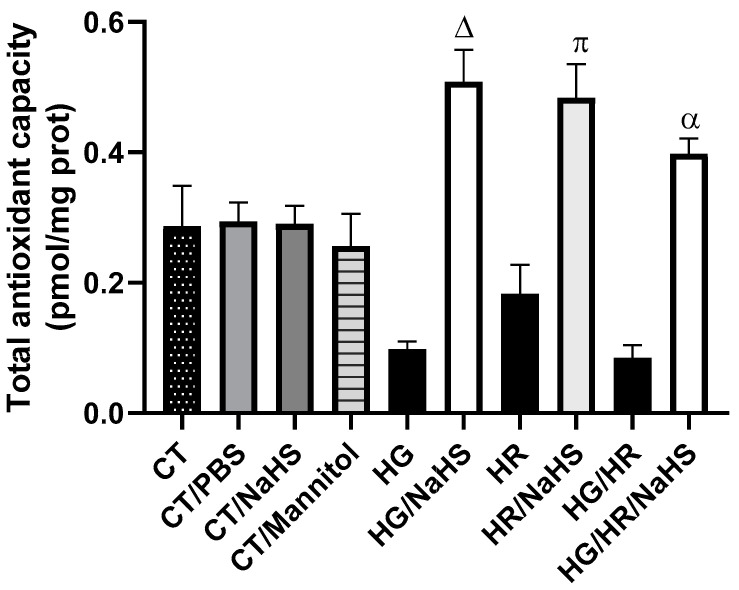
Total antioxidant capacity in primary cultures of cardiomyocytes incubated with NaHS (100 μM), exposed to HG, HR, or both conditions (HG/HR). Incubation with NaHS of cardiomyocytes exposed to HG, HR, or HG/HR increased total antioxidant capacity compared to their respective controls HG, HR, or HG/HR without NaHS. Δ = *p* < 0.05 vs. HG; π = *p* < 0.05 vs. HR; α = *p* < 0.05 vs. HG/HR. Two-way ANOVA followed by a Tukey post hoc test; *n* = 6.

The enzymatic and non-enzymatic antioxidant response to the different treatments was evaluated by measuring TAC in primary cultures of cardiomyocytes. Control cardiomyocytes incubated with PBS, NaHS, and mannitol showed similar TAC levels. Cardiomyocytes exposed to HG, HR, or HG/HR exhibited a decrease in TAC, and the incubation of these cardiomyocytes with NaHS reversed the decrease in TAC compared to their respective controls. These results suggest that NaHS promotes an antioxidant environment (Figure 4).

### 3.4. ROS Production

**Figure 5:**
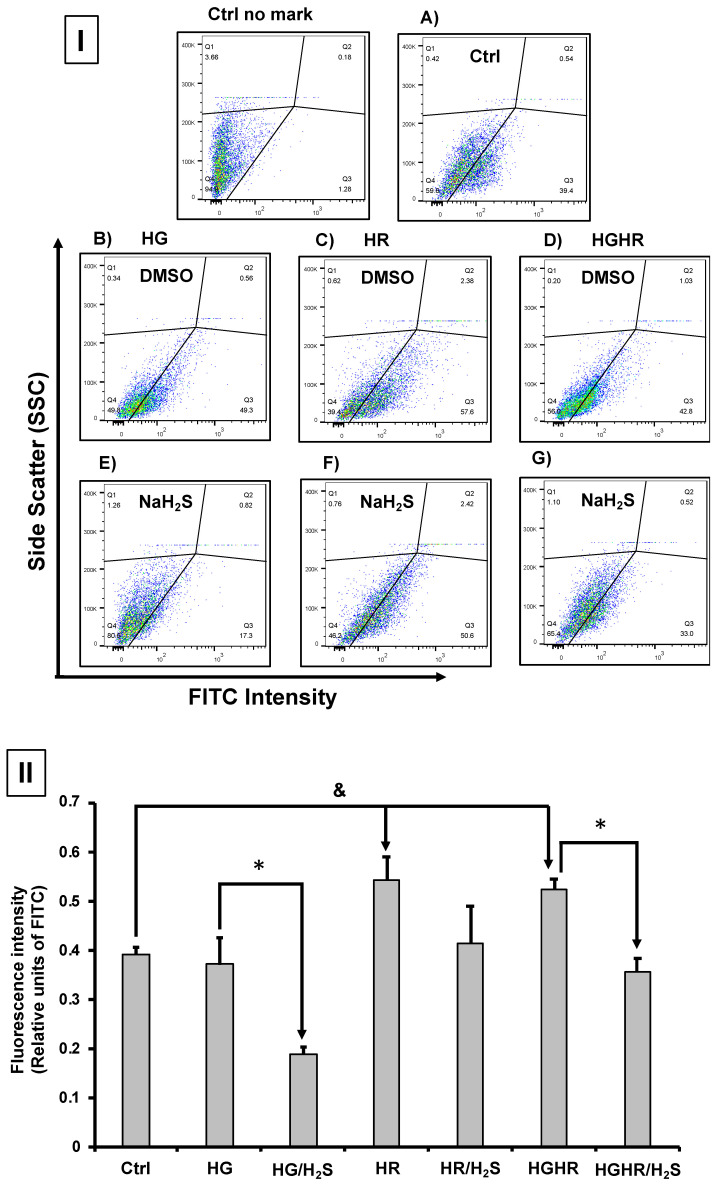
Evaluation of the production of reactive oxygen species (ROS) using the CellROX™ indicator and flow cytometry in primary cultures of cardiomyocytes. (**I**)**.** (**A**) Cardiomyocytes with CellROX only (control) and cardiomyocytes with CellROX exposed to (**B**) HG, (**C**) HR, and (**D**) HG/HR. In (**E**–**G**) ROS production decreased in cardiomyocytes exposed to HG and HG/HR incubated with NaHS (100 μM). (**II**) Fluorescence intensity taken from images in (**I**). & = *p* < 0.05 vs. Ctrl; * = *p* < 0.05 vs. HG and HG/HR. Two-way ANOVA followed by a Tukey post hoc test; *n* = 6.

CellROX™ Green reagent is a fluorogenic probe for measuring oxidative stress in cells. In our study, we assessed ROS production using the CellROX™ reagent and flow cytometry in primary cultures of cardiomyocytes cultures exposed to HG, HR, or HG/HR in the presence or absence of NaHS (100 μM). Primary cardiomyocyte cultures exposed to HR and HG/HR showed an increase in ROS production (represented by fluorescence intensity) (Figure 5I(C,D)), compared to the control group (Figure 5I(A),II). Treatment with NaHS (100 μM) in primary cardiomyocytes cultures exposed to HG and HG/HR conditions decreased ROS production (Figure 5I(E,G),II).

### 3.5. Evaluation of Oxidative Stress

**Figure 6:**
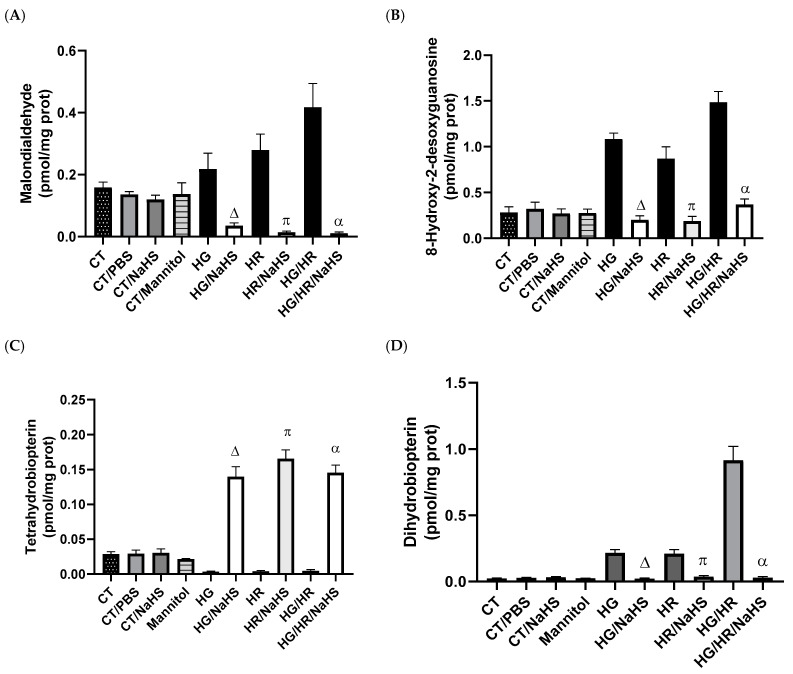
Evaluation of oxidative stress markers of the cofactor for endothelial nitric oxide synthase (eNOS), tetrahydrobiopterin (BH_4_), and its oxidation product, dihydrobiopterin (BH_2_) in primary cultures of cardiomyocytes incubated with NaHS (100 μM), and exposed to HG, HR, or both conditions (HG/HR). NaHS reversed the increase in oxidative stress marker concentrations: (**A**) malondialdehyde, (**B**) 8-hydroxy-2′-desoxguanosina; it also prevented the oxidation of (**C**) BH_4_ and therefore decreased the concentrations of its oxidation product (**D**) BH_2_. Δ = *p* < 0.05 vs. HG; π = *p* < 0.05 vs. HR; α = *p* < 0.05 vs. HG/HR. Two-way ANOVA followed by a Tukey post hoc test; *n* = 6.

To evaluate lipid peroxidation and oxidative DNA damage, we assessed malondialdehyde (MDA) and 8-hydroxy-2′-desoxyguanosine (8-OH-2dG) in primary cultures of cardiomyocytes exposed to HG, HR, or HG/HR. Control cardiomyocytes incubated with PBS, NaHS, and mannitol-maintained baseline values and did not show increased concentrations of MDA or 8-OH-2dG compared to control cardiomyocytes (CT). HG, HR, or HG/HR conditions in cardiomyocytes promoted an increase in MDA and 8-OH-2dG concentrations, and incubation with NaHS (100 μM) reversed the increase in these oxidative stress biomarkers (Figure 6A,B).

### 3.6. Evaluation of Cofactor for eNOS, Tetrahydrobiopterin (BH_4_), and Its Oxidation Product (BH_2_)

The coupling of eNOS requires the cofactor BH_4_ to produce nitric oxide (NO). The oxidation of BH_4_ generates BH_2_, promoting eNOS uncoupling and the generation of superoxide anion (O^2•−^) instead of NO. Control cardiomyocytes (CT) incubated with PBS, NaHS, and mannitol-maintained baseline concentrations of BH_4_ and BH_2_. The incubation with NaHS (100 μM) of cardiomyocytes exposed to HG, HR, or HG/HR promoted an increase in BH_4_ concentrations and a decrease in the concentrations of its oxidation product BH_2_ (Figure 6C,D). These results suggest that NaHS promotes eNOS coupling.

### 3.7. Evaluation of Palmitoyl-CoA Oxidase Activity and PPAR-α Expression

**Figure 7:**
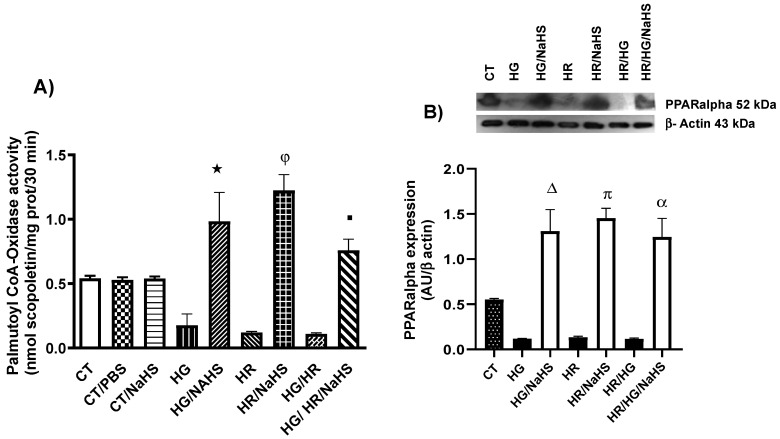
Evaluation of palmitoyl-CoA oxidase activity and PPAR-α expression in primary cultures of cardiomyocytes incubated with NaHS (100 μM), exposed to HG, HR, or both conditions (HG/HR). (**A**) Incubation with NaHS promoted an increase in PPAR-α expression under HG, HR, and HG/HR conditions. (**B**) Treatment with NaHS in cardiomyocytes exposed to HG, HR, and HG/HR conditions promoted an increase in PPAR-α expression. ★ = *p* < 0.05 vs. HG; φ = *p* < 0.05 vs. HR; ■ = *p* < 0.05 vs. HG/HR. Δ = *p* < 0.05 vs. HG; π = *p* < 0.05 vs. HR; α = *p* < 0.05 vs. HG/HR. Two-way ANOVA followed by a Tukey post hoc test; *n* = 6.

To evaluate PPAR-α expression in the different experimental groups, we performed a fluorometric study in which we measured palmitoyl-CoA oxidase activity. The experimental groups of cardiomyocytes exposed to HG, HR, and HG/HR conditions, and treated with NaHS, showed a reversal of the decrease in PPAR-α expression (Figure 7A). Furthermore, treatment with NaHS in cardiomyocytes exposed to HG, HR, and HG/HR conditions promoted an increase in PPAR-α expression (Figure 7B).

### 3.8. Evaluation of PPAR-γ, PGC-1α, AMPK, GLUT4, Keap1, Nrf2, and p-eNOS ^Ser1177^ Expression

**Figure 8:**
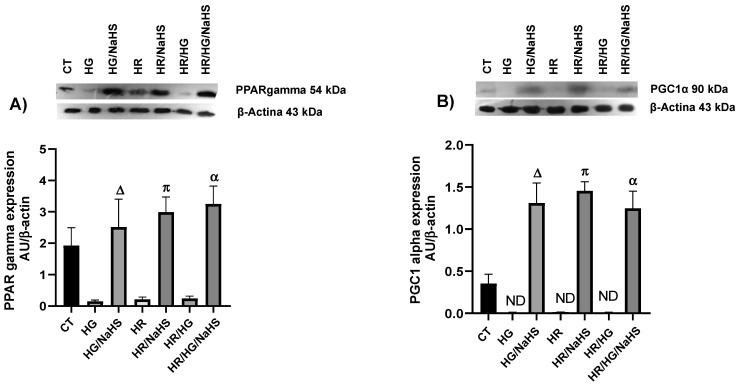

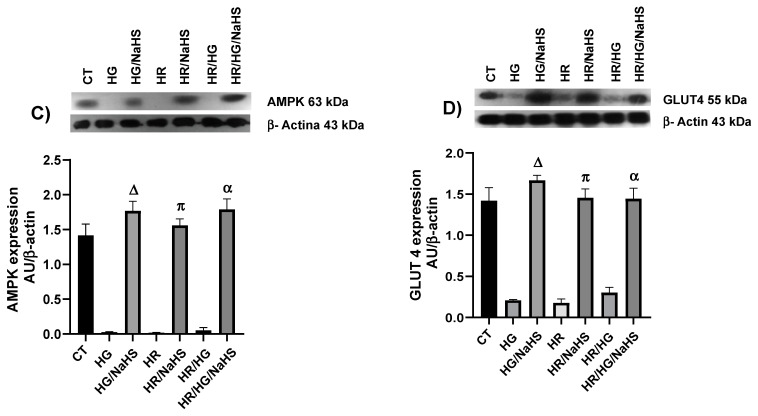
Expression of PPAR-γ, PGC 1α, AMPK, andGLUT4 in primary cultures of cardiomyocytes incubated with NaHS (100 μM), exposed to HG, HR, or both conditions (HG/HR). (**A**) Incubation with NaHS promoted an increase in PPAR-γ expression under HG, HR, and HG/HR conditions. (**B**) HG, HR, and HG/HR conditions promoted a decrease in PGC 1α expression. NaHS reversed the decrease in the expression of (**C**) AMPK and (**D**) GLUT4 under HG, HR, and HG/HR conditions. Δ = *p* < 0.05 vs. HG; π = *p* < 0.05 vs. HR; α = *p* < 0.05 vs. HG/HR. Two-way ANOVA followed by a Tukey post hoc test; *n* = 6.

**Figure 9:**
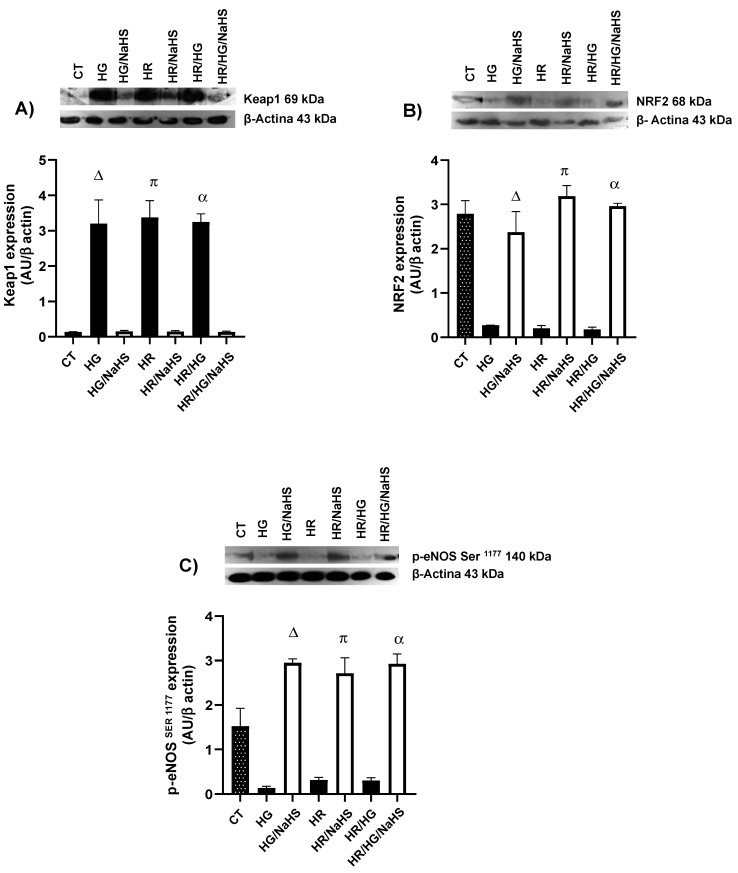
Expression of Keap1, Nrf2 y p-eNOS Ser^1177^ in primary cultures of cardiomyocytes incubated with NaHS (100 μM), exposed to HG, HR, or both conditions (HG/HR). (**A**) Incubation with NaHS promoted an increase in Keap 1 expression under HG, HR, and HG/HR conditions. (**B**) HG, HR, and HG/HR conditions promoted a decrease in Nrf2 expression. NaHS reversed the decrease in Nrf2 expression and (**C**) p-eNOS Ser^1177^ under HG, HR, and HG/HR conditions. Δ = *p* < 0.05 vs. HG; π = *p* < 0.05 vs. HR; α = *p* < 0.05 vs. HG/HR. Two-way ANOVA followed by a Tukey post hoc test; *n* = 3.

To evaluate whether the antioxidant environment generated by NaHS (100 μM) incubation in primary cultures of cardiomyocytes from different experimental groups involves the nuclear receptor PPAR-γ and its coactivator PGC-1α, we assessed the expression of these proteins using Western blot. We observed that NaHS promoted an increase in PPAR-γ and PGC-1α expression under HG, HR, and HG/HR compared to their respective controls (Figure 8A,B). Moreover, since PPAR-γ is related to carbohydrate metabolism, we assessed the expression of AMPK and GLUT4 and observed that treatment with NaHS in cardiomyocytes exposed to HG, HR, and HG/HR conditions reversed the decrease in these two proteins (Figure 8C,D). The transcription factor Nrf2 regulates the inducible expression of numerous antioxidant enzyme genes, and Nrf2 activity is constitutively repressed due to its binding to the cytoplasmic protein Keap1. Therefore, in our study, we assessed the expression of Keap1in cardiomyocytes exposed to HG, HR, and HG/HR. There was an increased expression of Keap1 compared to the experimental groups incubated with NaHS (Figure 9A). We also observed the incubation with this gasotransmitter in cardiomyocytes exposed to HG, HR, and HG/HR conditions reversed the decrease in the expression of transcription factor NRF2 (Figure 9B) and the enzyme p-eNOS ^Ser1177^ (Figure 9C) compared to their respective controls.

### 3.9. Evaluation of the Expression of SOD-Cu^2+^/Zn^2+^, SOD-Mn^2+^, Catalase, NOX4, and p47phox

**Figure 10:**
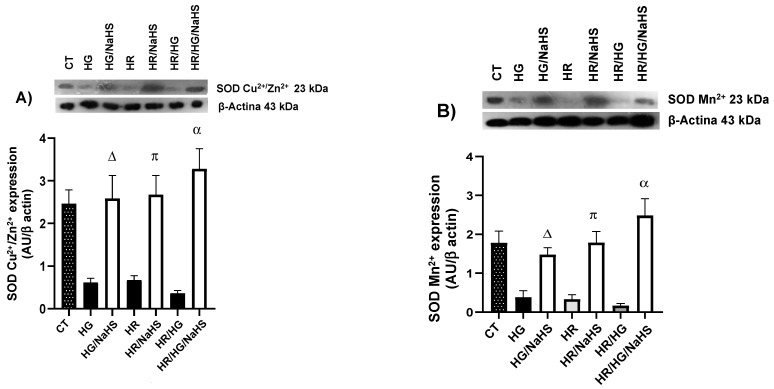

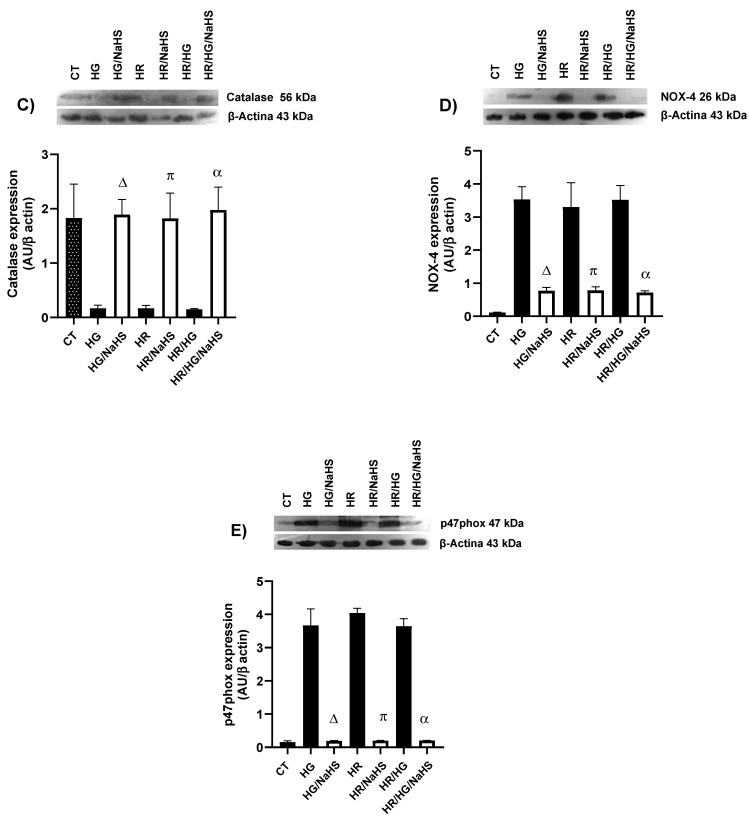
Expression superoxide dismutase-Cu^2+^/Zn^2+^ (SOD-Cu^2+^/Zn^2+^), SOD-Mn^2+^, catalase, NADPH oxidase 4 (NOX4), and p47phox in primary cultures of cardiomyocytes incubated with NaHS (100 μM), exposed to HG, HR, or both conditions (HG/HR). Incubation with NaHS under HG, HR, or HG/HR conditions promoted an increase in the expression of (**A**) SOD-Cu^2+^/Zn^2+^, (**B**) SOD-Mn^2+^, and (**C**) catalase and led to a decrease in the expression of (**D**) NOX4 and (**E**) p47phox. Δ = *p* < 0.05 vs. HG; π = *p* < 0.05 vs. HR; α = *p* < 0.05 vs. HG/HR. Two-way ANOVA followed by a Tukey post hoc test; *n* = 3.

To evaluate whether NaHS incubation in cardiomyocytes promotes an increase in the expression of certain antioxidant enzymes, we assessed the expression of SOD-Cu^2+^/Zn^2+^, SOD-Mn^2+^, and catalase using Western blot. In our study, we observed that NaHS promotes an increase in the expression of these antioxidant enzymes under HG, HR, and HG/HR conditions (Figure 10A–C) compared to their respective controls. Since NOX4 is a protein involved in ROS production and is regulated by its cytoplasmic subunit p47phox, we evaluated the expression of both proteins. Cardiomyocytes exposed to HG, HR, and HG/HR conditions and incubated with NaHS showed a decrease in the expression of NOX4 and p47phox compared to their respective controls (Figure 10D,E).

### 3.10. Evaluation of Mitochondrial Ultrastructure in Cardiomyocytes

**Figure 11:**
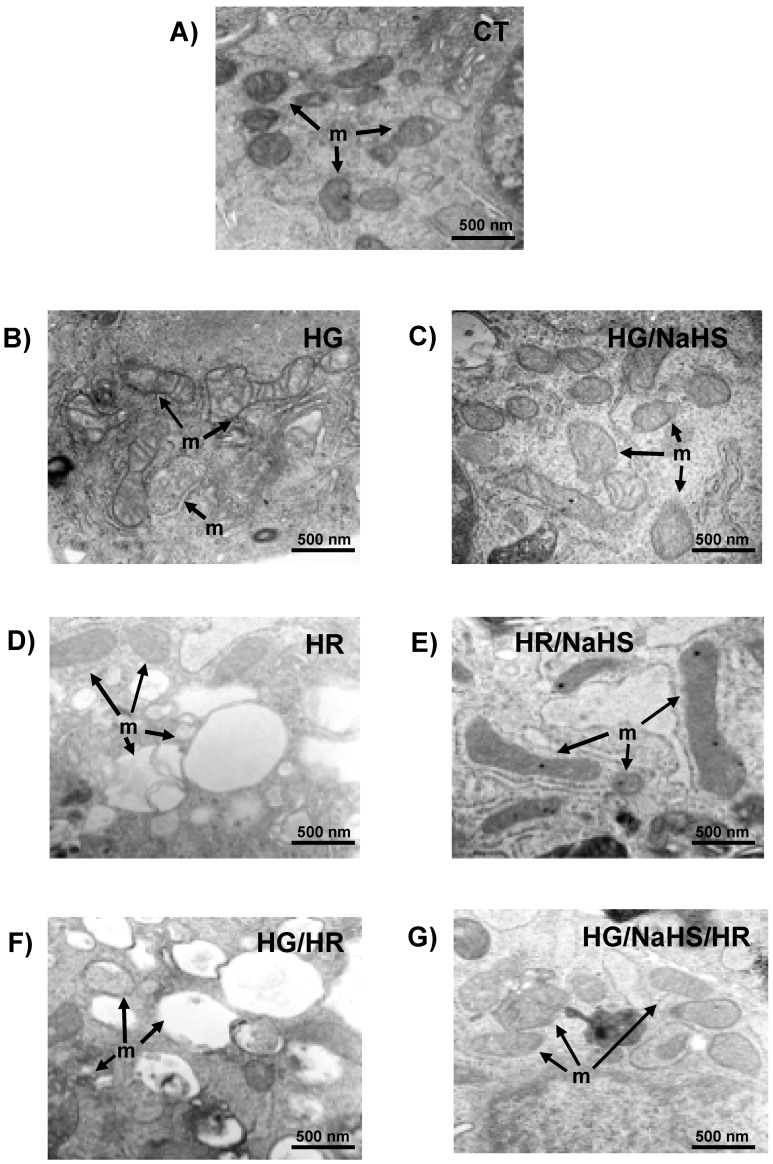
Evaluation of cardiomyocyte ultrastructure in cells incubated with NaHS (100 μM), exposed to HG, HR, or both conditions (HG/HR). NaHS treatment protects cardiomyocytes subjected to HG, HR, or both conditions from mitochondrial ultrastructure damage. (**A**) CT, (**B**) HG, (**C**) HG/NaHS (100 μM), (**D**) HR, (**E**) HR/NaHS (100 μM), (**F**) HG/HR, and (**G**) HG/HR/NaHS (100 μM). Arrows indicate mitochondria structures.

The effect of NaHS on the mitochondrial ultrastructure of cardiomyocytes subjected to hypoxia–reoxygenation, high glucose, and the combination of both (high glucose + hypoxia–reoxygenation) was evaluated. It is crucial that the mitochondrial structure is maintained, since this organelle is responsible for the synthesis of ATP necessary for cell survival. In Figure 11A, the micrograph of control cells is shown, where mitochondria with oval and elongated shapes are observed, as well as well-defined cristae, typical of a healthy and functioning mitochondrion.

When cardiomyocytes were exposed to high glucose (HG), the mitochondrial structure was compromised. In Figure 11B, circular mitochondria with internal vesicles, tubular and flat membranes, and swollen mitochondria, which appear completely round and broken, are seen. This structural damage to the mitochondria directly induced cell death. However, when NaHS was added to cardiomyocytes subjected to high glucose (HG/NaHS), protection against the HG-induced damage was observed, as seen in Figure 11C. The mitochondria had well-defined cristae and an oval structure, they no longer appeared swollen, and a greater number of dense mitochondria with intact cristae were visible, demonstrating the functionality of this organelle and the protective effect of NaHS.

When cardiomyocytes were exposed to hypoxia–reoxygenation (HR) (Figure 11D), significant damage to the mitochondrial structure was observed. As with high glucose, swollen and round mitochondria, as well as tubular and broken inner and outer membranes, were seen, indicating the loss of mitochondrial cristae and, consequently, mitochondrial dysfunction. In fact, under these conditions, there was a greater loss of cytochrome c, which would be related to cardiomyocyte death. Additionally, large vesicles were observed in the intracellular space. When the NaHS treatment was applied to these cells, the mitochondrial structure significantly improved even in the presence of hypoxia-reoxygenation (HR/NaHS). In Figure 11E, dense and elongated mitochondria with well-defined cristae are visible. Also, mitochondria undergoing fission were observed, which may indicate that NaHS treatment protects against HR-induced damage by activating this mitochondrial rescue pathway, ultimately benefiting cardiomyocyte survival.

When cardiomyocytes were subjected to both HG/HR conditions (Figure 11F), a significant increase in cellular damage was observed, particularly the loss of mitochondrial structure. A higher number of broken mitochondria was seen, and the ones that were visible were less dense and round; many were empty without an internal structure, indicating the loss of mitochondrial membranes and, consequently, the loss of their function. In contrast, when the cells were incubated with NaHS, an improvement in mitochondrial structure was observed despite being exposed to HG/HR conditions (Figure 11G). A greater number of intact mitochondria with well-defined cristae and their typical oval and elongated shape were visible, indicating that NaHS protects the cardiomyocyte from HG/HR-induced damage.

## 4. Discussion

Oxidative stress is an imbalance between the antioxidant defense systems and the production of ROS. ROS cause lipid peroxidation, protein oxidation, and DNA damage, thereby altering normal cellular functions. Myocardial ischemia/reperfusion (I/R) injury occurs when blood supply is interrupted (ischemia) and then restored (reperfusion), leading to an “explosion” of ROS produced by the mitochondria [31]. Mitochondrial ROS are generated by electron leakage from the electron transport chain, resulting in the incomplete reduction of oxygen to the superoxide anion (O_2_^•−^). Additionally, reverse electron transport driven by succinate leads to the production of O_2_^•−^ in the mitochondrial matrix from complex I during reperfusion. Mitochondrial NOX4 also contributes to H_2_O_2_ generation and the restoration of pH, along with mitochondrial calcium overload and excessive ROS generation, and it causes the formation of the mitochondrial permeability transition pore (mPTP) after reperfusion, leading to cardiomyocyte death [32]. Oxidative stress induced by hyperglycemia in T2D plays an important role in complications and dysfunction of many vital organs, such as the heart, kidneys, nerves, and eyes [33]. Specifically, hyperglycemia damages endothelial cells, and the close link between diabetes and early vascular disease is well established [34]. In our study, we used primary cardiomyocyte cultures from neonatal rats and subjected them to hyperglycemia (HG), HR, or both conditions to mimic the damage caused by high glucose concentrations and I/R. We observed that these conditions decreased cell viability (Figure 3), reduced total antioxidant capacity (Figure 4), increased ROS production (Figure 5), raised oxidative damage biomarkers (Figure 6A,B), increased NOX4 and p47phox expression (Figure 10D,E), and induced damage to the mitochondrial ultrastructural (Figure 11B,D,F).

After the discoveries of nitric oxide (NO) and carbon monoxide (CO), hydrogen sulfide (H_2_S) was identified as the third important gasotransmitter [35]. H_2_S can be produced in most tissues of the human body through both enzymatic and non-enzymatic pathways. The most important enzymatic pathways use L-cysteine as a substrate and require one of three specific enzymes: cystathionine γ-lyase (CSE), cystathionine β-synthase (CBS), and 3-mercaptopyruvate sulfotransferase [36]. Oxidative stress is increased, eNOS activity is decreased, and NO levels are reduced in the heart and liver in mice lacking CSE, which exacerbates I/R-induced damage [37]. In our study, we observed that treatment with NaHS in primary cardiomyocytes subjected to hyperglycemia, HR, or both conditions increased BH_4_ levels (Figure 6C) and decreased BH_2_ levels (Figure 6D), thus promoting eNOS coupling.

H_2_S has a variety of important physiological functions in mammalian tissues, and it helps protect cells against apoptosis and oxidative stress [35]. Zhong et al. reported that NaHS treatment (a H_2_S donor) of primary neonatal rat cardiomyocyte cultures with high glucose significantly decreased ROS levels and increased NO levels [38]. NaHS also protects against cardiomyocyte apoptosis induced by high glucose concentrations by attenuating oxidative stress and altering the expression of apoptosis-regulating genes [35]. Resembling these studies, we observed that primary cardiomyocyte cultures subjected to hyperglycemia, HR, or both conditions and treated with NaHS increased total antioxidant capacity (Figure 4) and decreased ROS production (Figure 5), leading to a reduction in lipid peroxidation (Figure 6A) and oxidative damage to the DNA (Figure 6B).

Pretreatment of neonatal rat cardiomyocytes with NaHS reduced ROS levels during HR conditions by inhibiting the activity of mitochondrial complex IV and increasing SOD activity, including SOD-Cu^2+^/Zn^2+^ and SOD-Mn^2+^. In addition, H_2_S binds to the catalytic Cu-center of Cu^2+^, Zn^2+^ SOD and is a genuine substrate of the enzyme. Whether this reaction plays a physiological role in H_2_S scavenging is still under investigation [39].

The antioxidant defense system provides critical protection of the biological system by limiting the harmful effects of ROS. There are many antioxidant enzymes, including SOD, GPx, glutathione reductase, catalase, etc. [40]. In addition to the enzymatic antioxidants, non-enzymatic antioxidants (uric acid, bilirubin, reduced glutathione (GSH), melatonin, etc.) also play an important role in maintaining normal ROS levels [41]. Under various pathological conditions, including T2D and I/R injury, redox balance may be altered, leading to negative consequences for the cell [42]. H_2_S exerts antioxidant effects through various mechanisms, reducing levels of ROS and reactive nitrogen species (RNS), modulating cellular GSH and thioredoxin (Trx-1) levels, or increasing the expression of antioxidant enzymes by activating Nrf2 [43]. In our study, the NaHS treatment in primary cardiomyocyte cultures subjected to hyperglycemia, HR, or both conditions promoted an increase in the expression of Nrf2 (Figure 9C) and a decrease in the expression of its regulator Keap1 (Figure 9B). Hassan MI et al. demonstrated that a putative antioxidant-responsive element (ARE) with which Nrf2 can interact has been identified in the promoter/upstream sequences of the CSE gene in some species, suggesting that Nrf2 might upregulate CSE expression [44]. Moreover, H_2_S donors can also upregulate CSE expression via Nrf2 activation. In addition to transcription factor binding regulation, CSE expression can also be regulated by promoter methylation [45]. H_2_S exerts antioxidant effects by increasing the expression of antioxidant enzymes [43], and, in our study, we observed an increase in the expression of antioxidant proteins SOD-Cu^2+^/Zn^2+^, SOD-Mn^2+^, and catalase (Figure 9A–C), similar to what has been reported, which was associated with increased cell viability (Figure 3), decreased ROS (Figure 5I,II), and improved mitochondrial ultrastructure (Figure 11).

H_2_S protects the cardiovascular and cerebrovascular systems by reducing inflammation and dilating blood vessels, generating significant interest in H_2_S-based therapeutic strategies [46]. Liang et al. reported that exogenous NaHS had a protective effect against myocardial mitochondrial injury in sepsis induced by Cecal ligation puncture in mice. The observed effect involved the PPAR-γ coactivator-1 alpha (PGC-1α)/Nrf2 and mitochondrial biosynthesis pathway [47]. In our research, we observed a reversal of the decreased expression of PPAR-γ and PGC-1α in primary cardiomyocyte cultures subjected to hyperglycemia, HR, or both conditions (Figure 8A,B), resembling what has been reported. Moreover, PPAR-γ activation increases the expression and translocation of glucose transporters GLUT1 and GLUT4, thus increasing glucose uptake in the liver and skeletal muscle cells, reducing plasma glucose levels [48]. In our study, we observed a reversal of the decreased expression of GLUT4 (Figure 8D). Therefore, our results suggest that H_2_S promotes an increase in the expression of PPAR-γ/GLUT4, which could improve glucose uptake, a mechanism of great importance for patients with hyperglycemia.

PGC-1α plays an important role in gene transcription through its interaction with PPARs, including PPAR-α [11]. PPAR-α agonists, including fenofibrate and WY14643, activate AMP-activated protein kinase (AMPK), which can phosphorylate and activate eNOS [49]. Lin et al. reported that H_2_S protected endothelial cells from hyperglycemia-induced damage by activating the PI3K/Akt/eNOS pathway [50]. Similar to these studies, we observed that treatment with NaHS in primary neonatal rat cardiomyocytes subjected to hyperglycemia, HR, or both conditions increased the activity and expression of PPAR-α (Figure 7A,B) as well as eNOS expression (Figure 9C). Additionally, we observed a reversal in the decrease of AMPK expression (Figure 8C). The relationship between H_2_S and PPAR-α has not been reported in cardiomyocytes; however, in a human hepatoma cell line (HepG2), NaHS increased the expression of ATP-binding cassette transporter A1 (ABCA1) by promoting the nuclear translocation of PPAR-α, providing a fundamental mechanism for H_2_S’s anti-atherogenic activity since ABCA1 mediates reverse cholesterol transport [51].

PPAR-α and its coactivator PGC-1α are critical factors in mitochondrial biogenesis through the activation of mitochondrial transcription factors and various nuclear transcription factors (Nrf1, Nrf2) [11]. NaHS may exert a protective effect against doxorubicin-induced cardiotoxicity by inhibiting ferroptosis through the antioxidant pathway solute carrier family 7-member 11/glutathione/glutathione peroxidase 4 (SLC7A11/GSH/GPx4) which depends on Keap1/Nrf2 [52]. Wang et al. reported that NaHS treatment in db/db mice with type 2 diabetes (T2D) increased Keap1 ubiquitination by preserving its E3 ligase synoviolin (Syvn1), resulting in the nuclear translocation of Nrf2. Therefore, NaHS activates the Nrf2/GPx4/GSH pathway, suppressing ferroptosis and decreasing mitochondrial apoptosis [53]. Importantly, in our study, we observed an increase in the expression and activity of PPAR-α (Figure 7A,B), a reversal in the decrease of Nrf2 expression (Figure 9B), and a decrease in the expression of the regulator of the transcription factor, Keap1 (Figure 9A), which could be related to the increase in antioxidant capacity and the observed cytoprotective effect.

## 5. Conclusions

The treatment with NaHS generated a cytoprotective effect in primary cultures of cardiomyocytes subjected to hyperglycemia, HR, or both conditions, promoting an increase in the expression and activity of PPAR-α. Moreover, treatment with this gasotransmitter led to an increase in the expression of PPAR-γ/PGC-1α/AMPK/GLUT4/Nrf2/p-eNOS/CAT/SOD and a decrease in ROS production and oxidative stress biomarkers.

## Data Availability

The authors confirm that the data supporting the findings of this study are available within the article. The datasets of this study are available from corresponding author upon reasonable request.

## Abbreviations

HR, hypoxia–reoxygenation; HG, high glucose; ROS, reactive oxygen species; PPARs, peroxisome proliferator-activated receptors; SOD, superoxide dismutase; Cat, catalase; eNOS, endothelial nitric oxide synthase.
